# Peer Review of “Development and Assessment of a Point-of-Care Application (Genomic Medicine Guidance) for Heritable Thoracic Aortic Disease”

**DOI:** 10.2196/64356

**Published:** 2024-10-08

**Authors:** Joseph Walsh

**Affiliations:** 1 University of Victoria, Victoria, BC, Canada

**Keywords:** genomic medicine, point of care, thoracic aortic aneurysm, aortic dissection, decision support

*This is a peer-review report for “Development and Assessment of a Point-of-Care Application (Genomic Medicine Guidance) for Heritable Thoracic Aortic Disease.”*

## Round 1 Review

### General Comments

This paper [1] describes the development and implementation of a novel application designed to assist clinicians and patients in managing heritable thoracic aortic diseases (HTADs) through accessible genomic information. The Genomic Medicine Guidance application, developed using REDCap, integrates genetic data with clinical recommendations to provide comprehensive guidance on diagnosis, treatment, and surveillance of HTAD. Preliminary user feedback indicates high usability and positive impact on clinical guidance, suggesting the Genomic Medicine Guidance application could significantly contribute to personalized patient care and potentially influence clinical practices toward better management of HTAD.

This manuscript is well structured, presenting a clear problem statement, detailed development methodology, results from initial user feedback, and a discussion on the implications of the application in clinical settings. It addresses a critical gap in the application of genomic medicine in clinical practice, particularly in the management of heritable aortic diseases. Overall, the manuscript presents a strong case for further research and development.

### Specific Comments

#### Major Comments

1. The article would benefit from a more detailed analysis of user feedback, including data on the application’s impact on clinical decision-making and patient outcomes.
2. Consider including possibilities for future updates and challenges in a broader implementation.

## Round 2 Review

### General Comments

Thank you for the updated manuscript. My previous comments have been addressed.

## Abbreviations

HTAD: heritable thoracic aortic disease

## References

[1] Patil R, Ashraf F, Abu Dayeh S, Prakash SK (2024). Development and Assessment of a Point-of-Care Application (Genomic Medicine Guidance) for Heritable Thoracic Aortic Disease. JMIRx Med.

